# Evaluation of *WISP1* as a candidate gene for bone mineral density in the Old Order Amish

**DOI:** 10.1038/s41598-018-25272-4

**Published:** 2018-05-08

**Authors:** Xing Wang, Shabnam Salimi, Zhongliang Deng, James Perry, Kathleen A. Ryan, Zhizhen Li, Dongfang Liu, Elizabeth Streeten, Alan R. Shuldiner, Mao Fu

**Affiliations:** 1 School of Medicine, Division of Endocrinology, Diabetes and Nutrition, and Program for Personalized and Genomic Medicine, University of Maryland School of Medicine, Baltimore, MD 21201 USA; 2 Department of Orthopedic Surgery, the Second Affiliated Hospital of Chongqing Medical University, Chongqing, 400010 China; 3 The Department of Endocrinology, the Second Affiliated Hospital of Chongqing Medical University, Chongqing, 400010 China

## Abstract

Wnt1-inducible signaling pathway protein-1 (*WISP1*) is a novel target of the Wnt pathway for modulating osteogenesis and improving bone strength. However, it is not clear if genetic variants in the *WISP1* region are associated with bone mineral density (BMD) in human. The aim of this study is to investigate the role of genetic variation in *WISP1* gene as a determinant of BMD in 1,510 Old Order Amish (OOA). We performed regional association analysis of 58 tag variants within 5 kb upstream and downstream to *WISP1* with BMD and found 5 variants that were associated with BMD at multiple skeletal sites (*P* values from 2.89 × 10^−6^ to 1.62 × 10^−2^), with some significant associations even after adjustment for multiple comparisons. To replicate these results in an independent dataset, we performed a look-up of BMD associations with these variants in European ancestry subjects from the large GEFOS Consortium and observed the nominal associations of two of these variants with BMD (*P* values: 0.031 to 0.048). In conclusion, we have demonstrated that genetic variants surrounding *WISP1* are associated with BMD at multiple skeletal sites in the OOA, thus influencing osteoporosis risk. These results support a role for the *WISP1* gene on influencing variation in BMD.

## Introduction

Osteoporosis is a common disorder affecting hundreds of millions of people and is one of the leading causes of fractures in the world. This disease accounts for approximately 1.5 million new fracture cases each year, representing a huge economic burden on health care systems, with annual costs estimated to be $17 billion in the United States alone and expected to rise 50% by the year 2025^1,2^. Osteoporosis is mainly characterized by bone fragility (most individuals have a low BMD) and it is a highly heritable trait with heritability ranging from 0.5 to 0.8^3^. Recent studies have shown that multiple genetic variants in *Wnt* pathway components including *AXIN1*, *CTNNB1*, *DKK1*, *LRP4*, *LRP5*, *MEF2C*, *RSPO3*, *SFRP4*, *SOST*, *WLS*, *WNT3*, *WNT4*, *WNT5B* and *WNT16* are associated with BMD^4–11^. Identifying new genetic variants that influence BMD could lead to new strategies to treat osteoporosis.

Within the components of the *Wnt* signaling pathway, WISP1, also known as CCN4, has been found to be a novel target for modulating osteogenesis and improving bone strength. The *WISP1* gene is located on human chromosome 8q24.22 and contains six exons and five introns. Expression of *WISP1* has been observed in the developing skeleton and later in both osteoblast precursors and osteoblastic cells^12^, specifically at sites of new bone formation during development or fracture healing^13^. Moreover, mice with *WISP1* knocked out have lower BMD, trabecular bone volume/total volume, and cortical bone thickness than wild-type mice^14^, and transgenic mice with human *WISP1* over-expressed have increased BMD, trabecular thickness, and bone volume/total volume over wild-type controls^15^. In humans, genetic variants of *WISP1* have been associated with increasing the risk of these diseases, such as spinal osteoarthritis, scirrhous gastric carcinoma, lung cancer, and myocardial infarction^16–19^. These findings indicate that *WISP1* is necessary for bone formation and regulation of skeletogenesis. Therefore, we hypothesized that polymorphic changes around *WISP1* loci are associated with BMD.

The aim of this study was to evaluate the role of polymorphisms around *WISP1* locus on BMD at total hip, hip femoral neck, hip intertrochanter, hip trochanter and lumbar spine in 1,510 OOA individuals.

## Results

### Clinical characteristics of BMD measures and other anthropometrical traits

This study recruited 1,510 OOA who were measured for BMD using Dual-energy X-ray Absorptiometry (DXA). Participant clinical characteristics were shown in Table 1. This study included 715 males (mean age 50.73 years, range 20–95 years) and 795 females (mean age 51.90 years, range 18–93 years). There was no difference in mean age between males and females. On average, females showed higher body mass index (BMI) than males (28.65 ± 5.73 kg/m^2^ vs 26.66 ± 4.01 kg/m^2^, *p* < 0.001). However, the mean levels of BMD in the hip total, femoral neck, hip intertrochanter, trochanter and lumbar spine were significantly higher in males than in females (*p* < 0.001).

**Table 1:** Clinical characteristics of the study population (mean ± SD).

	Male	Female	*p*
AGE(year)	50.73 ± 15.26	51.9 ± 14.65	0.192
BMI(kg/m^2^)	26.66 ± 4.01	28.65 ± 5.73	1.70 × 10^−14^
TH-BMD(g/cm^2^)	1.02 ± 0.13	0.93 ± 0.15	4.93 × 10^−31^
FN-BMD(g/cm^2^)	0.86 ± 0.13	0.82 ± 0.14	7.77 × 10^−8^
HIT-BMD(g/cm^2^)	1.19 ± 0.16	1.09 ± 0.18	1.09 × 10^−27^
HTC-BMD(g/cm^2^)	0.78 ± 0.11	0.71 ± 0.12	5.39 × 10^−35^
LS-BMD(g/cm^2^)	0.97 ± 0.14	0.93 ± 0.16	4.32 × 10^−8^

TH-BMD: total hip BMD; HFN-BMD: hip femoral neck BMD; HIT-BMD: hip intertrochanter BMD; HTC-BMD: hip trochanter BMD; LS-BMD: lumbar spine BMD; *p*: *p* value; SD: standard deviation.

### Heritability of the BMD at multiple skeletal sites in the OOA

The heritability of each of the densitometric phenotypes was shown in Table 2 and was based on the most parsimonious model of variance component analysis for each phenotype, including only significant sources of variation. All of the results were statistically significant (*p* < 0.05). In the whole group consisting of males and females, the heritabilities of the BMD measurements in specific sites were high with variations from 0.66 (lumbar spine BMD) to 0.58 (hip femoral neck BMD). In the sex-stratified model, we found that heritabilities of BMD at multiple skeleton sites were generally greater in females than in males. The heritability of hip intertrochanter BMD (h^2^ = 0.56) was the highest in the male group, whereas the heritability of lumbar spine BMD (h^2^ = 0.67) was the highest in female group.

**Table 2:** Heritability of BMD in the OOA.

Skeletal BMD	Overall		Male		Female	
	h^2^ ± SE	h^2^_*p*	h^2^ ± SE	h^2^_*p*	h^2^ ± SE	h^2^_*p*
TH-BMD	0.62 ± 0.05	1.96 × 10^−39^	0.53 ± 0.09	1.65 × 10^−8^	0.61 ± 0.09	2.08 × 10^−16^
HFN-BMD	0.58 ± 0.06	3.66 × 10^−32^	0.55 ± 0.09	3.12 × 10^−8^	0.59 ± 0.09	2.11 × 10^−13^
HIT-BMD	0.62 ± 0.05	2.72 × 10^−39^	0.56 ± 0.09	6.51 × 10^−9^	0.59 ± 0.09	8.07 × 10^−16^
HTC-BMD	0.61 ± 0.06	4.87 × 10^−38^	0.52 ± 0.09	3.03 × 10^−9^	0.56 ± 0.09	1.12 × 10^−14^
LS-BMD	0.66 ± 0.05	9.20 × 10^−39^	0.51 ± 0.09	7.23 × 10^−10^	0.67 ± 0.09	1.61 × 10^−16^

TH-BMD: total hip BMD; HIT-BMD: hip intertrochanter BMD; HFN-BMD: hip femoral neck BMD; HTC-BMD: hip trochanter BMD; LS-BMD: lumbar spine BMD; SE: standard error of h^2^; p: p value, Covariance including study, sex, age and family structure.

### Association of variants in *WISP1* with BMD in the OOA

**Figure 1:**
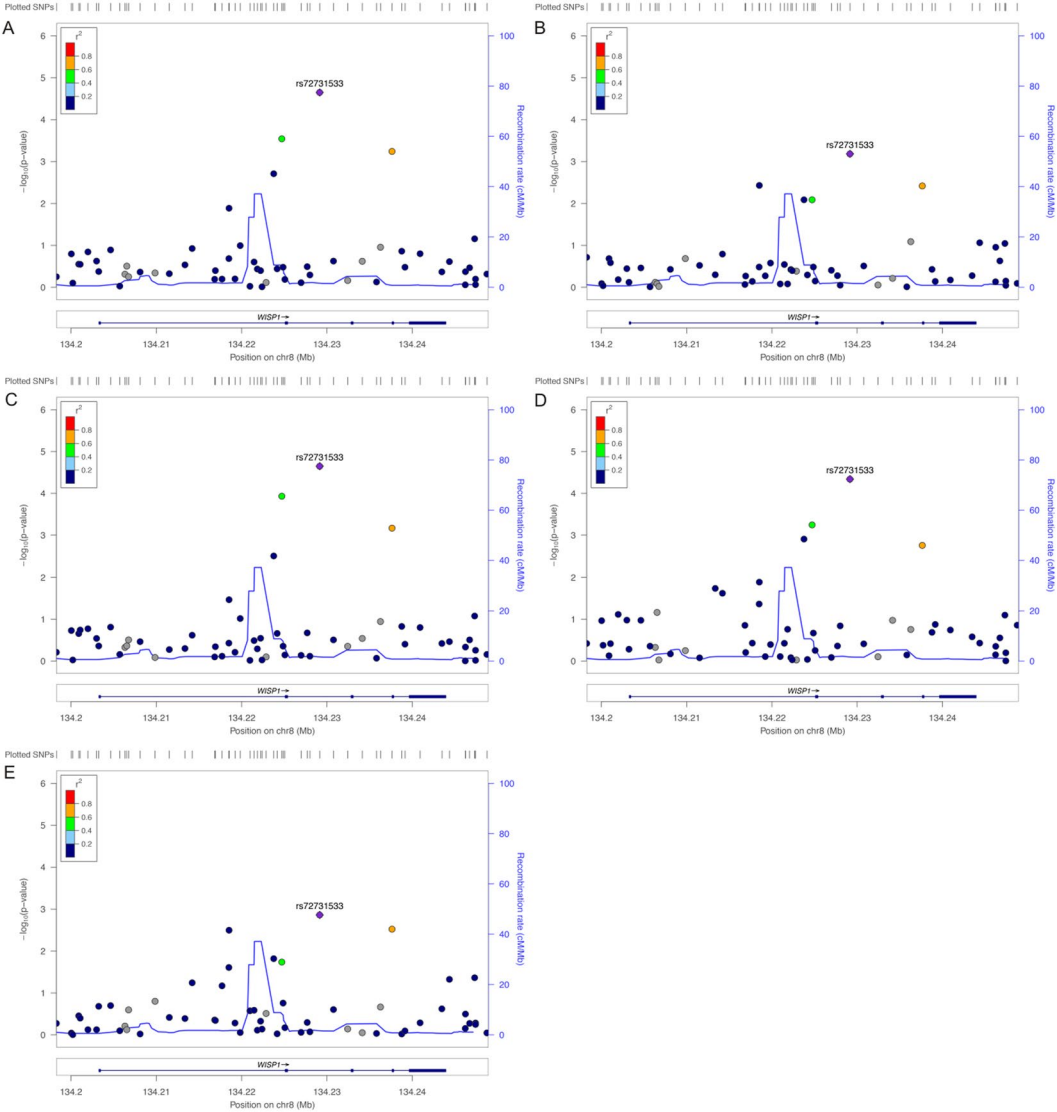
Regional association plots for the *WISP1* (+/−5 kb) region for BMD in (**A**) total hip, (**B**) femoral neck, (**C**) hip intertrochanter, (**D**) hip trochanter and (**E**) lumbar spine. Genetic variants in and around the *WISP1* gene (+/−5 kb) are depicted on x axis, and the corresponding association *p* value (−log10) on y axis. The top SNP, i.e. rs72731533, is denoted by a purple color. Variants are color coded according to their LD (r^2^) with the lead SNP (1000 Genomes Project Nov 2014 EUR population). The recombination rate (grey line) and position of gene, its exons and direction of transcription are also indicated.

We performed association analyses of 58 tag variants in *WISP1* gene region with BMD in total hip, femoral neck, hip intertrochanter, hip trochanter and lumbar spine, adjusted for family structure, study, age, sex, age*sex (model 1). We found 5 variants that were significantly associated with all BMD traits (*P* values range from 2.89 × 10^−6^ to 1.62 × 10^−2^, Table 3 and Fig. 1). The SNP (rs72731533) located in intron 2 was the most significantly associated variant with all the phenotypes (total hip BMD, *p* = 3.85 × 10^−6^; hip femoral neck BMD, *p* = 1.66 × 10^−4^; hip intertrochanter BMD, *p* = 2.89 × 10^−6^; hip trochanter BMD, *p* = 1.67 × 10^−5^ and lumbar spine BMD, *p* = 7.84 × 10^−4^; Table 3 and Fig. 1). After Bonferroni correction for multiple testing (*p* < 1.72 × 10^−4^), rs72731533 was still significantly associated with total hip, intertrochanter and trochanter BMD (Table 3 and Fig. 1). To determine if *WISP1* influencing variation in BMD was independent of BMI, we adjusted BMI as a covariant (model 2) and found that these variants remained significant association with BMD traits (Table 3).

**Table 3:** Association results of variants surrounding *WISP1* gene with BMD traits.

RSNUM	POS	Alleles	Location	MAF^a^	MAF^b^	INFO	TRAIT	Model 1		Model 2		Meta-analysis	
		RA/EA						β ± SE	*p*	β ± SE	*p*	β ± SE	*p*
rs116873248	134218542	C/T	intronic	0.11	0.07	0.84	TH-BMD	0.16 ± 0.06	5.13 × 10^−3^	0.10 ± 0.05	4.35 × 10^−2^		
							HFN-BMD	0.19 ± 0.06	1.60 × 10^−3^	0.13 ± 0.05	1.28 × 10^−2^	0.02 ± 0.01	0.191
							HIT-BMD	0.15 ± 0.06	1.20 × 10^−2^	0.09 ± 0.05	8.83 × 10^−2^		
							HTC-BMD	0.16 ± 0.06	7.22 × 10^−3^	0.10 ± 0.05	5.18 × 10^−2^		
							LS-BMD	0.20 ± 0.06	1.63 × 10^−3^	0.16 ± 0.06	7.54 × 10^−2^	0.036 ± 0.02	3.10 × 10^−2^
rs7824539	134224717	C/T	intronic	0.12	0.15	0.92	TH-BMD	0.22 ± 0.06	6.30 × 10^−5^	0.17 ± 0.05	2.87 × 10^−4^		
							HFN-BMD	0.16 ± 0.06	3.79 × 10^−3^	0.12 ± 0.05	1.57 × 10^−2^	0.01 ± 0.01	0.404
							HIT-BMD	0.25 ± 0.06	1.30 × 10^−5^	0.20 ± 0.05	5.16 × 10^−5^		
							HTC-BMD	0.20 ± 0.06	3.29 × 10^−4^	0.16 ± 0.05	1.64 × 10^−3^		
							LS-BMD	0.14 ± 0.06	1.62 × 10^−2^	0.11 ± 0.06	4.44 × 10^−2^	0.005 ± 0.01	0.748
rs11778573	134228930	T/G	intronic	0.45	0.43	0.99	TH-BMD	0.10 ± 0.04	5.33 × 10^−3^	0.09 ± 0.03	2.35 × 10^−3^		
							HFN-BMD	0.10 ± 0.04	5.40 × 10^−3^	0.09 ± 0.03	3.02 × 10^−3^	0.02 ± 0.01	4.82 × 10^−2^
							HIT-BMD	0.09 ± 0.04	1.44 × 10^−2^	0.08 ± 0.03	8.93 × 10^−3^		
							HTC-BMD	0.11 ± 0.04	2.45 × 10^−3^	0.10 ± 0.03	1.20 × 10^−3^		
							LS-BMD	0.12 ± 0.04	1.77 × 10^−3^	0.12 ± 0.04	1.59 × 10^−3^	0.019 ± 0.01	3.20 × 10^−2^
rs72731533	134229152	C/G	intronic	0.08	0.05	0.98	TH-BMD	0.29 ± 0.06	3.85 × 10^−6^	0.21 ± 0.05	8.15 × 10^−5^		
							HFN-BMD	0.24 ± 0.06	1.66 × 10^−4^	0.17 ± 0.06	2.64 × 10^−3^	0.01 ± 0.02	0.661
							HIT-BMD	0.30 ± 0.06	2.89 × 10^−6^	0.22 ± 0.06	5.94 × 10^−5^		
							HTC-BMD	0.28 ± 0.06	1.67 × 10^−5^	0.20 ± 0.06	3.65 × 10^−4^		
							LS-BMD	0.23 ± 0.07	7.48 × 10^−4^	0.18 ± 0.06	6.43 × 10^−3^	−0.026 ± 0.02	0.223
rs35513885	134237635	G/T	exonic	0.1	0.04	0.93	TH-BMD	0.22 ± 0.06	2.19 × 10^−4^	0.13 ± 0.05	1.23 × 10^−2^		
							HFN-BMD	0.19 ± 0.06	1.68 × 10^−3^	0.11 ± 0.05	4.63 × 10^−2^	0.01 ± 0.02	0.658
							HIT-BMD	0.23 ± 0.06	1.77 × 10^−4^	0.14 ± 0.05	9.65 × 10^−3^		
							HTC-BMD	0.20 ± 0.06	1.08 × 10^−3^	0.11 ± 0.05	3.66 × 10^−2^		
							LS-BMD	0.20 ± 0.06	1.72 × 10^−3^	0.14 ± 0.06	2.20 × 10^−2^	−0.031 ± 0.02	0.148

TH-BMD: total hip BMD; HIT-BMD: hip intertrochanter BMD; HFN-BMD: hip femoral neck BMD; HTC-BMD: hip trochanter BMD; LS-BMD: lumbar spine BMD; POS: position in the genome based on GRCh37.p13 Annotation Release 105; INFO: imputation quality score; RA: reference allele; EA: effect allele; MAF^a^: minor allele frequency in the OOA; MAF^b^: minor allele frequency from meta-analysis of GEFOS.seq project; β: estimates of effect size expressed as adjusted SD per copy of the effect allele; SE: standard error of β; *p*: p value; Model 1: covariance including study, sex, age, and family structure; Model 2: covariance including INVNORM, age, sex, age*sex, family structure and BMI; Meta-analysis: 2015 meta-analyses of whole-genome sequencing, whole-exome sequencing, and deep imputation of genotype data devoted by GEnetic Factors for OSteoporosis (GEFOS) Consortium.

### Replication study in GEFOS

We further looked for associations of the 5 lead SNPs in the published meta-analysis data in Caucasian^20^. We found that one SNP (rs11778573) were nominally associated with BMD at both femoral neck (*p* = 4.82 × 10^−2^) and lumbar spine (*p* = 3.20 × 10^−2^), and another SNP (rs116873248) showed suggestive level of association with BMD at lumbar spine (*p* = 3.10 × 10^−2^) (Table 3). We compared the minor allele frequencies of these 5 polymorphisms in the OOA with minor allele frequencies in the GEFOS Caucasian population and found that OOA minor allele frequencies varied little from GEFOS Caucasian population allele frequencies (Table 3).

### Conditional analyses

**Figure 2:**
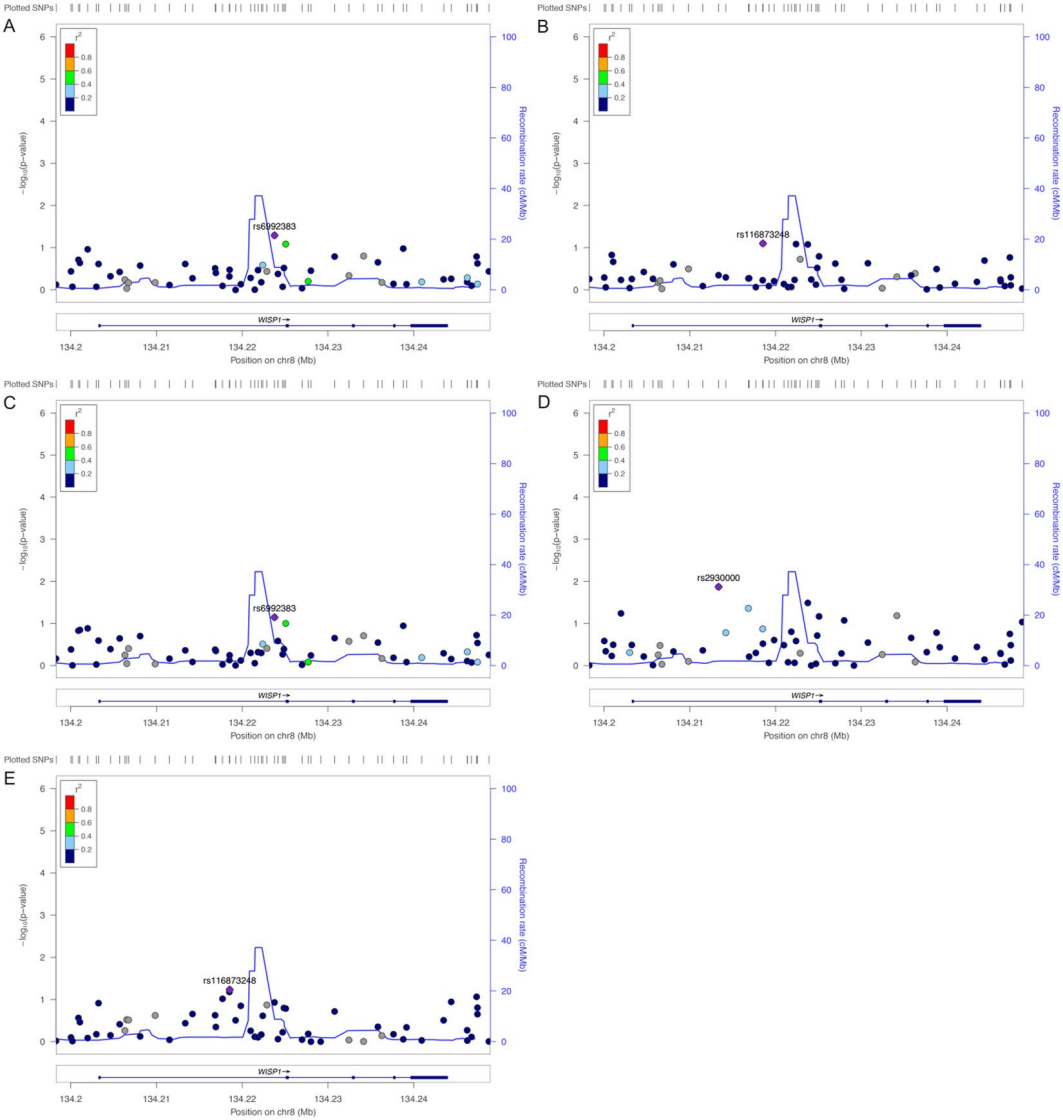
Conditional association plots for the *WISP1* (+/−5 kb) region for BMD in (**A**) total hip, (**B**) femoral neck, (**C**) hip intertrochanter, (**D**) hip trochanter and (**E**) lumbar spine. Genetic variants within the *WISP1* (+/−5 kb) are depicted (x axis) along with their association *p* value (−log10). The top SNPs, i.e. rs6992383, are denoted by a purple color. Variants are color coded according to their LD (r^2^) with the lead SNP (1000 Genomes Project Nov 2014 EUR population). The recombination rate (grey line) and position of gene, its exons and direction of transcription are also indicated.

**Figure 3:**
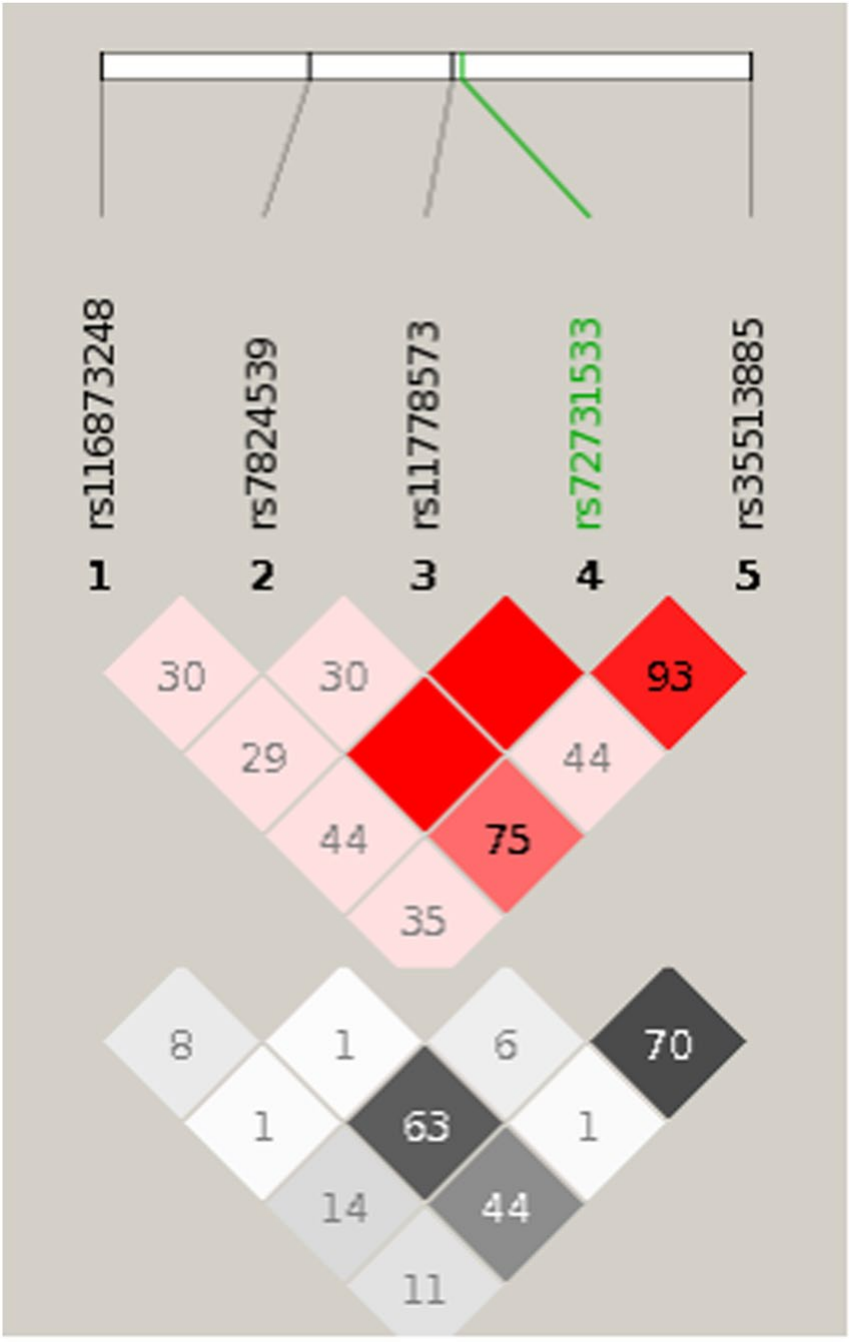
Linkage disequilibrium(D’ top, r^2^ bottom) structure for all associated SNPs. For both (D’ and r^2^, values range from 0 (no LD) to 100 (complete LD). The thick white line represents a strand of a chromosome. The black bars on the white line of the chromosome are SNPs that have been identified and sequenced. These SNP locations are labeled in this picture from 1 to 5. Each of these SNPs has a name with corresponding numeric code. Each SNP is represented by a labeled grey triangle below the thick white line. Value in each box indicates (D’ or r^2^ value between 2 SNPs (intersection). For (D’ figures, no value indicates complete LD (100).

To further determine independent variants, we performed a conditional analysis using the top variant rs72731533 as a covariate and no signal in this region remained (*p* > 0.01) (Fig. 2). This result indicated that only one association signal in this region was associated with BMD at multiple skeletal sites. We performed linkage disequilibrium (LD) analysis for the five variants associated with BMD using Haploview and found that rs35513885 (exon 4, A118S) was in high LD with rs72731533 (r^2^ = 0.70, Fig. 3). Using rs35513885 as a covariate, conditional association analysis showed that rs72731533 was still associated with BMD traits (Table 4). This result suggested that rs72731533 may be a leading variant to regulate BMD.

**Table 4:** Conditional association analysis using rs35513885 and rs72731533.

TRAIT	rs72731533 adjusted for rs35513885		rs35513885 adjusted for rs72731533	
	β ± SE	*p*	β ± SE	*p*
TH-BMD	0.05 ± 0.02	3.31 × 10^−3^	−0.01 ± 0.02	0.691
HFN-BMD	0.03 ± 0.02	3.24 × 10^−2^	0.001 ± 0.02	0.974
HIT-BMD	0.06 ± 0.02	2.43 × 10^−3^	−0.01 ± 0.02	0.617
HTC-BMD	0.04 ± 0.01	2.39 × 10^−3^	−0.01 ± 0.01	0.462
LS-BMD	0.03 ± 0.02	0.165	0.01 ± 0.02	0.567

TH-BMD: total hip BMD; HFN-BMD: hip femoral neck BMD; HIT-BMD: hip intertrochanter BMD; HTC-BMD: hip trochanter BMD; LS-BMD: lumbar spine BMD; β: estimates of effect size expressed as adjusted SD per copy of the effect allele; SE: standard error of β; *p*: p-value in the conditional analyses.

### Bioinformatics analysis

**Figure 4:**
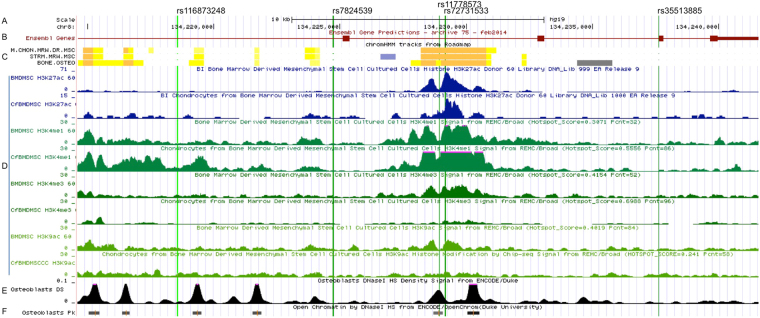
UCSC bioinformatics view of the *WISP1* region (chr8:134214753–134241422, GRCh37/hg19). The light green, long vertical line indicates the position of SNPs. (**A**) The Base Position feature; (**B**) The *WISP1* gene prediction using Ensembl gene prediction; (**C**) the chromatin state segmentation for the cell types using the imputed ChromHMM (Hidden Markov Model), orange: active enhancer, yellow: weak enhancer or enhancer acetylation, PaleTurquoise: Het (Heterochromatin), Silver: ReprPC (Repressed PolyComb); (**D**) four histone markers modification map in two cell lines generated by Broad Institute using ChIP-Seq. (**E**) shows the density signal of DNaseI HS and (**F**) shows the peaks of DNaseI HS in the osteoblast from ENCODE/OpenChrom(Duke University). HMM (Hidden Markov Model), STRM.MRW.MSC and BMDMSC (Bone Marrow Derived Mesenchymal Stem Cell Cultured Cells; M.CHON.MRW.DR.MSC and cfBMDMSC (Chondrocytes from Bone Marrow Derived Mesenchymal Stem Cell Cultured Cells); BONE.OSTEO (primary osteoblast); DS (density signal); PK (peaks).

Based on the UCSC Genome Bioinformatics website (http://genome.ucsc.edu/), we annotated all five BMD association variants in regulatory elements catalogued in Encyclopedia of DNA Elements (ENCODE) project. As shown in Fig. 4C, rs11778573 and rs72731533 are situated near the active enhancer elements on STRM.MRW.MSC (Bone Marrow Derived Mesenchymal Stem Cell Cultured Cells), M.CHON.MRW.DR.MSC (Chondrocytes from Bone Marrow Derived Mesenchymal Stem Cell Cultured Cells) and BONE.OSTEO (primary osteoblast). As shown in Fig. 4D, rs72731533 is situated near the enhancer elements (H3K4Me1 and H3K27Ac marks) and also near the promoter elements (H3K4Me3 and H3K9Ac marks) on the BMDMSC (Bone Marrow Derived Mesenchymal Stem Cell Cultured Cells) and cfBMDMSC (Chondrocytes from Bone Marrow Derived Mesenchymal Stem Cell Cultured Cells) from ENCODE, which indicated that rs72731533 was probably involved in the regulation of gene expression. The SNP, rs72731533, also fell into a DNase Hypersensitivity site in primary osteoblasts (Fig. 4E,F). The accessible chromatin zone is functionally related to transcriptional activity, since this remodeled state is necessary for the binding of proteins such as transcription factors. We further examined whether the 5 significant association SNPs influence gene expression using public databases such as the Genotype-Tissue Expression (GTEx) project and ExSNP. However, we did not find significant eQTL for these SNPs that may be due to lack of bone tissue information in the databases. We further conduct cis-eQTL analyses on the SNPs in *WISP1* gene region with transcripts in primary osteoblasts (obtained from bone biopsies)^21^. We found that a SNP, rs144161059, in high LD (linkage disequilibrium) with rs72731533 (D’ = 1) was significantly associated with *WISP1* gene expression (*P* = 2.17 × 10^−3^). The SNP, rs144161059, with low minor allele frequency (MAF = 0.003 in 1000 Genomes) failed to be imputed in this study.

## Discussion

The *Wnt* signaling pathway is one of the most important signaling pathways in bone regulation because of its essential role in development, particularly in tissue specification and in cellular migration^22^. This signaling pathway influences all types of bone cells (osteoblasts, osteoclasts and osteocytes) and has showed to be important in skeletal development, maintenance of skeletal homeostasis and in bone remodeling^23^. Recently, several independent studies, with the goal to detect candidate genes underlying osteoporosis, revealed that many genes in the *Wnt* signal pathway are associated with lumbar spine, hip femoral neck and whole body BMD, bone strength, cortical bone thickness, and fracture risk^10,24^. Within the components of the *Wnt* signaling pathway, the gene coding for WISP1 has been found as a novel target for modulating osteogenesis and improving bone strength. The importance of *WISP1* gene in the regulation of BMD and bone strength had been also confirmed by *WISP1* knockout (*WISP1*^*−/−*^) mice^14^ and human *WISP1* transgenic mice^15^. In this study, we have investigated whether the variants in the *WISP1* gene are associated with BMD at multiple skeletal sites in the OOA. Our finding is the first report that shows the significant association of the variant surrounding *WISP1* gene on BMD in the OOA.

The heritability of BMD is significantly high (h^2^ >0.5) reported by several studies [6, 17, 27, 30]. We calculated heritability of BMD at multiple skeletal sites in 1,510 OOA subjects and compared the difference of heritability between males and females. The heritability of lumbar spine BMD (h^2^ = 0.66) was the highest and the heritability of hip femoral neck BMD (h^2^ = 0.58) was the lowest in the whole group of males and females combined suggesting a greater genetic determinant of BMD in the lumbar spine than in proximal femur that was similar to previous studies^25^. Variance in the BMD heritability of different skeletal sites is possibly due to dissimilar external forces placed on certain bones of the skeleton. Previous studies had successfully noted site-specific variations of BMD heritability that are gender-dependent^26^. In this study, we observed that both female and male groups have strong genetic correlations of BMD and we found that heritability of BMD was partly different between females (h^2^:0.59–0.69) and males (h^2^:0.53–0.56). It was similar to our previous result that the heritability in BMD was larger in women than in men^27,28^. However, our results do not support some previous studies that males tend to have higher heritability values than females^29,30^. Amish males perform farm work and Amish females perform housekeeping work. There is higher physical activity in Amish males than in Amish females that may contribute the difference of heritability in BMD. Tse and colleagues observed that the high degree of BMD heritability may reflect a preservation of genetic influence in the relative absence of external forces^26^. Our results revealed that 53–69% of variance in BMD of OOA is contributed by genetic factors.

*WISP1* is a member of the CCN family growth factors and its variants have been confirmed to be associated with various pathologies. Tao J. *et al*.^17^ found that the *WISP1* rs16893344 variant allele (T) was associated with a significantly increased risk of myocardial infarction. Chen J. *et al*.^19^ found that *WISP1* rs16893344, rs2977530, rs2977537, and rs62514004 (P < 0.05) polymorphisms were related to susceptibility of lung cancer; and *WISP1* rs11778573, rs16893344, rs2977536, rs2977549 and rs62514004 polymorphisms were significantly associated with platinum-based chemotherapy response in lung cancer patients. Interestingly, Tomohiko *et al*.^16^ evaluated the association of a SNP (rs2929970) in the *WISP1* 3′-UTR region with the presence of osteophytes, endplate sclerosis, and narrowing of disc spaces for spinal osteoarthritis in 304 postmenopausal Japanese women and found strong associations of rs2929970 with endplate sclerosis. Several GWAS identified BMD SNPs are nominally associated with prevalent radiographic knee osteoarthritis (OA)^31^. The previous studies suggested that the loci, associated with osteoarthritis, might be also association with BMD. In the present study, we examined the association between polymorphisms around the *WISP1* gene and BMD at the total hip, femoral neck, hip intertrochanter, hip trochanter and lumbar spine to investigate a possible contribution of *WISP1* to human bone metabolism.

In this study, we identified 58 tag genetic variations with a minor allele frequency (MAF) of at least 1% surrounding the *WISP1* gene (+/−5 kb) and found 5 variants significantly associated with all BMD traits. The top associated variant was a SNP (rs72731533) that was located in intron 2 of the *WISP1* gene. We further investigated whether the 5 SNPs are independently associated with BMD and found that only one association signal in this region is associated with BMD at multiple skeletal sites. A previous study reported that an intergenetic SNP, rs7839059, located on chromosome 8q24.12 near to *WISP1* gene was significantly associated with cortical vBMD (*P* = 1.2 × 10^−10^)^32^. We found that rs7839059 was associated with all the phenotypes (total hip BMD, *p* = 5.32 × 10^−5^; hip femoral neck BMD, *p* = 2.88 × 10^−4^; hip intertrochanter BMD, *p* = 1.52 × 10^−4^; hip trochanter BMD, *p* = 6.06 × 10^−5^ and lumbar spine BMD, *p* = 3.53 × 10^−2^). We performed a conditional analysis that showed rs7839059 was independent of the associated signal in *WIPS1* gene (Table 5). To replicate our results in larger sample sets, we checked the published meta-analysis data in Caucasians^20^ and found that two SNPs (rs11778573 and rs116873248) were nominally associated with BMD at multiple skeleton sites (*p* = 0.031–0.0482). We think the following two reasons may cause nominal replications. First, we used chips for genotyping that just included common variants. The significantly associated variants may be partially link to causal variants. Second, the significantly associated variants may be population specific. The most significant SNP, rs72731533, was involved in regulatory elements (such as enhancer and promoter around *WISP1* gene) in both MSCs and OPCs. We did not find significant eQTL for rs72731533. However, the SNP, rs144161059, in high LD with rs72731533 was significantly associated with *WISP1* gene expression in primary osteoblasts, although, we did not imputed rs144161059 in this study. Thus, a denser fine-mapping study using sequencing of the *WISP1* locus will provide a better resolution to identify potential causal variant(s) and will be helpful for future functional validation. Those reports suggested that variants around the *WISP1* region were actively involved in regulation of multiple phenotypes. This combined evidence suggests that polymorphisms around the *WISP1* are associated with BMD at multiple skeletal sites.

**Table 5:** Conditional association analysis using rs7839059 and rs72731533.

TRAIT	rs72731533 adjusted for rs7839059		rs7839059 adjusted for rs72731533	
	β ± SE	*p*	β ± SE	*p*
TH-BMD	−0.02 ± 0.01	6.95 × 10^−4^	0.04 ± 0.01	3.31 × 10^−5^
HFN-BMD	−0.02 ± 0.01	1.54 × 10^−3^	0.03 ± 0.01	5.37 × 10^−4^
HIT-BMD	−0.03 ± 0.01	1.74 × 10^−3^	0.05 ± 0.01	2.81 × 10^−5^
HTC-BMD	−0.02 ± 0.01	7.03 × 10^−4^	0.03 ± 0.01	7.89 × 10^−5^
LS-BMD	−0.01 ± 0.01	0.358	0.03 ± 0.01	1.05 × 10^−3^

TH-BMD: total hip BMD; HFN-BMD: hip femoral neck BMD; HIT-BMD: hip intertrochanter BMD; HTC-BMD: hip trochanter BMD; LS-BMD: lumbar spine BMD; β: estimates of effect size expressed as adjusted SD per copy of the effect allele; SE: standard error of β; *p*: p-value in the conditional analyses.

In conclusion, we performed a regional analysis for 5 kb upstream and downstream *WISP1* with specific BMD adjusted for study, sex and age in 1,510 OOA. We confirmed that genetic variation at the *WISP1* locus is significantly associated with BMD at multiple skeletal sites. These results identify that genetic variants in *WISP1* gene region are associated with BMD levels. Bioinformatics analyses suggest that this feasible association is partly caused by regulatory effects on the enhancer or promoter of *WISP1* gene. The results suggest that *WISP1* gene could be important for bone health in humans, as has already been shown *in vitro* and *vivo*. The denser fine-mapping, replication, and functional validation will be necessary to understand the mechanisms underlying these associations.

## Methods

### Subjects

The OOA of Lancaster Pennsylvania are relatively homogenous in terms of both genetic ancestry and lifestyle characteristics. Subjects (*n* = 1,510) included in this study were adults aged 18 years or older, who participated in the Amish Family Osteoporosis Study (AFOS), the Amish Family Longevity Study (AFLS) and Pharmacogenomics of Anti-Platelet Intervention (PAPI). The protocols and procedures for those studies were approved by the Institutional Review Boards of the University of Maryland and all subjects gave written informed consent. All methods were performed in accordance with the relevant guidelines and regulations. Details of these studies design, recruitment, and phenotyping had been described previously^33–36^. Briefly, the AFOS was started in March 1997 to study genes that are important for bone health. This was a population-based study to identify individuals with osteoporosis. After the identification of individuals with osteoporosis by BMD, family members were recruited including first-degree relatives aged 20 years and older. Spouses were also invited to participate in the study. The goal of AFLS is to identify genes related to a long and healthy life and to understand the function of these genes. The goal of the PAPI study was to understand the reason why some people do not respond to commonly used medications to prevent myocardial infarction, including aspirin and clopidogrel (Plavix). The subjects in the three studies had bone mineral density (BMD) measured by DXA.

### Body composition and bone mineral density

Examinations were conducted at the Amish Research Clinic in Strasburg, PA^31,33,37,38^. Height was measured by using a stadiometer. Height and weight were recorded without shoes. Body mass index (BMI) was calculated as weight in kilograms divided by height in meters squared. Areal BMD (grams per square centimeter) was measured by DXA at the total hip, hip femoral neck, hip intertrochanter, hip trochanter bone and lumbar spine (L1–L4), using a Hologic 4500 W (Bedford, MA, USA) by a technician certified in bone densitometry. Our study focused on multiple densitometric phenotypes that we considered clinically relevant. For femoral neck, there were 1510 OOA examined (male n = 715 and female n = 795), but for spine, five subjects were excluded, due to either prior spine surgery or structural abnormalities, leaving male n = 713 and female n = 792 participants.

### Genotyping and single nucleotide polymorphism (SNP) Selection

Study participants were genotyped using either the Affymetrix 500k or Affymetrix v6.0 SNP chips by the Genomics Core Laboratory at the University of Maryland. SNPs with a minor allele frequency (MAF) ≥1%, a call rate exceeding 95% and conforming to the expectations of Hardy-Weinberg equilibrium (*p* > 10^−6^) were used for imputation with IMPUTE-2 using 1000 G CEU reference sample phase2. SNPs with Imputation quality score (INFO) ≥30% were considered. Selection of the tagSNPs was performed based on the OOA genotyping data. Using the aggressive tagger mode of Haploview version 4.2 (http://www.broadinstitute.org/haploview/), we selected 58 tagSNPs which cover all common genetic variation within 5 kb upstream and downstream to *WISP1* gene (Chr8: 134198282–134248933, GRCh37.p13) (Table 6). Association analysis including these 58 tagSNPs was performed.

**Table 6:** Overview of the selected tag SNPs.

TagSNP	SNPs(r^2^>=0.8)
rs7844966	rs11783250, rs13263504, rs13279370, rs3739261, rs7840551, rs7844272, rs7844423, rs7844482, rs7844513
rs201323658	rs57455867, rs12156037, rs16893344, rs16904845, rs55884058, rs60282000, rs62514004, rs72731505, rs72731508
rs3834380	rs2280834, rs2929966, rs2929967, rs2929970, rs2929972, rs2977548, rs2977549, rs2977551, rs2977553
rs10089461	rs10956696, rs11774069, rs11774084, rs11780866, rs17713284, rs2272645, rs28615068, rs28637383, rs28669728
rs7005834	rs10100792, rs11777304, rs11777380, rs11781004, rs13250295, rs13259044, rs13281186, rs7006080
rs72731555	rs112894423, rs199804336, rs201030002, rs72731540, rs72731542, rs72731543, rs72731545, rs72731553
rs10094601	rs2004891, rs2977527, rs2977530, rs4382455, rs72731515, rs72731516, rs753722
rs1109563	rs2977537, rs62514011, rs62514012, rs62514013, rs873873
rs6992383	rs11778573, rs4354288, rs6982341, rs7826828, rs7828685
rs2929934	rs2929937, rs2929946, rs2929947, rs753723
rs33995880	rs16904853, rs35784897, rs78162004
rs754958	rs2929969, rs2929971, rs2929973
rs2930000	rs2977525, rs2977529
rs2929986	rs2977522, rs2977523
rs142107754	rs16893349, rs185865626
rs12548174	rs7842500
rs3739262	rs7007905
rs34386977	rs61330647
rs10108233	rs4330674
rs12164193	rs34282673
rs2977533	rs2977536
rs1078778	rs2977531
rs200747824	rs2977519
rs7843546	rs2013158
rs199697351	rs147194671
rs7824539	
rs72731533	
rs72731528	
rs72731507	
rs71299054	
rs6471115	
rs62514033	
rs62514029	
rs35513885	
rs2977555	
rs2977552	
rs2977520	
rs2929975	
rs2929965	
rs2013146	
rs188829590	
rs186242527	
rs17634696	
rs147211333	
rs146930369	
rs146643864	
rs146626818	
rs143261612	
rs141788195	
rs141351177	
rs13261909	
rs13254146	
rs12155747	
rs11774368	
rs116873248	
rs115857620	
rs10956697	
rs10092372	

### Statistical Analysis

Summary statistics of baseline clinical characteristics were expressed as unadjusted means ± standard deviations (SD) using the SPSS statistics version 23 (IBM Corporation, N.Y., NY, USA). The association analyses were carried out using in-house software called MMAP (https://mmap.github.io/). The polygenic component was modeled using the relationship matrix derived from the complete 14-generation Amish pedigree structure. We included family structure, study, age, sex, age*sex, as covariates in the association models. BMI was associated with BMD on univariate analysis and was therefore included as a covariate in model 2. Subgroup analyses to determine whether there were differences in gender were performed. Estimation of the additive genetic heritability follows basic quantitative genetic theory, which models the phenotypic covariance (conditional upon covariate effects) between two individuals in a pedigree as a function of their degree of biologic relatedness. Maximum likelihood methods were used to estimate the values of the parameters, such as heritability, that resulted in highest likelihood obtained across all of the pedigrees. Covariates for BMD heritability analysis were study, sex and age. *P*-values less than 0.05 were considered as significant. Correction for multiple testing was performed using the Bonferroni method for the number of SNPs and traits tested (*P* = 0.05/(58 × 5) = 1.72 × 10^−4^).

### Bioinformatics analysis

Analysis of linkage disequilibrium (LD) statistics (*r*^2^) surrounding variants of interest was performed using Haploview version 4.2 (http://www.broadinstitute.org/haploview/). Prediction of histone marks and DNAse hypersensitivity sites was performed using HaploReg v4.1^39^, and the five SNPs were annotated in regulatory elements cataloged in Encyclopedia of DNA Elements (ENCODE) project according to UCSC Genome Bioinformatics website (http://genome.ucsc.edu/). The eQTL analyses were performed in GTEx (https://www.gtexportal.org/home/), ExSNP (http://www.exsnp.org/), and primary osteoblasts (obtained from bone biopsies)^21^.
